# Dietary Carbohydrates and Lipids in the Pathogenesis of Leaky Gut Syndrome: An Overview

**DOI:** 10.3390/ijms21218368

**Published:** 2020-11-08

**Authors:** Agata Binienda, Agata Twardowska, Adam Makaro, Maciej Salaga

**Affiliations:** Department of Biochemistry, Faculty of Medicine, Medical University of Lodz, 92-215 Lodz, Poland; agata.binienda@gmail.com (A.B.); agata.twardowska@stud.umed.lodz.pl (A.T.); adammakaro@gmail.com (A.M.)

**Keywords:** leaky gut syndrome, lipids, carbohydrates

## Abstract

This review summarizes the recent knowledge on the effects of dietary carbohydrates and lipids on the pathophysiology of leaky gut syndrome (LGS). Alterations in intestinal barrier permeability may lead to serious gastrointestinal (GI) disorders. LGS is caused by intestinal hyperpermeability due to changes in the expression levels and functioning of tight junctions. The influence of dietary habits on intestinal physiology is clearly visible in incidence rates of intestinal diseases in industrial and developing countries. Diseases which are linked to intestinal hyperpermeability tend to localize to Westernized countries, where a diet rich in fats and refined carbohydrates predominates. Several studies suggest that fructose is one of the key carbohydrates involved in the regulation of the intestinal permeability and its overuse may cause harmful effects, such as tight junction protein dysfunction. On the other hand, short chain fatty acids (mainly butyrate) at appropriate concentrations may lead to the reduction of intestinal permeability, which is beneficial in LGS. However, long chain fatty acids, including *n*-3 and *n*-6 polyunsaturated fatty acids have unclear properties. Some of those behave as components untightening and tightening the intestinal membrane.

## 1. Introduction

### 1.1. The Structure of The Intestinal Barrier

In humans, the intestinal barrier covers a surface of about 400 m^2^ and forms the largest interface with the external environment [1]. It prevents penetration of microorganisms, toxins and antigens through the intestinal wall and loss of water with electrolytes while allowing nutrients adsorption and waste secretion. To maintain these features, intestinal barrier consists of both physical, chemical and biological components including mucus, epithelial cells sealed through tight junctions (TJs), immune cells and intestinal microbiota [2]. 

Mucus is the first defense line, which precludes adhesion and permeation of microorganisms and toxins through the intestinal wall thereby prevents the development of inflammation [3]. Besides water, mucus is composed of both secreted (Muc2, Muc5, Muc6) and membrane bound (Muc1, Muc3, Muc4, Muc17) mucins. Of note, Muc2 glycoprotein plays an essential role in epithelial protection since Muc2 knockout mice spontaneously develop severe colon inflammation and inflammation-induced colorectal cancer [4,5]. The structure of mucus differs depending on the region of gastrointestinal (GI) tract. In the small intestine, the single mucus layer forms a diffusion barrier abundant in antimicrobial substances such as defensins, lysozyme and IgA produced in response to bacteria or their toxins by Paneth cells [3]. As the mucus is not attached to the intestinal wall it continually moves with peristaltic waves transporting bacteria down to the colon [6]. In the large intestine, mucus is organized into two layers: inner and outer. The inner layer is impervious to bacteria and forms a barrier that separates microorganisms from the epithelium. It is also responsible for rehydration, regeneration and acts as a shield against digestive enzymes [7]. Just as in the small intestine, the outer mucus layer of the colon is less dense and unattached to the intestinal wall. In contrast to inner mucus layer, which is sterile, the outer mucus layer is inhabited by bacteria, especially *Bacterioides acidifaciens, Bacterioides fragilis* and *Akkermansia muciniphila,* which contribute to maintaining intestinal barrier function mostly by limiting growth of pathogenic strains and regulating biochemical pathways important in preserving the structure and function of the GI tract [8]. There are several examples in the literature demonstrating diverse effects of bacteria and their metabolites on intestinal barrier integrity [9]. For instance, *A. muciniphilia* regulates ileal production of antimicrobial peptide RegIIIγ, which exhibits bactericidal activity against Gram-positive bacteria. By increasing expression of RegIIIγ *A.muciniphilia* not only promotes its own survival via decreased competition for resources but also reduces the opportunity for development of pathological strains [10]. Additionally, *Bacterioides theraiotaomicron* enhances expression of the small proline-rich protein 2A, which is responsible for stabilization of desmosomes at the epithelial villus [11]. Modification of intestinal microbiota composition may result in decreased expression of TJ proteins, impaired mucus production and secretion or increase in production of proinflammatory cytokines and consequently induced intestinal dysbiosis. Intestinal dysbiosis is characterized by expansion of phatobionts such as *E. coli* and loss of commensals which results in decreased microbial diversity. Major alterations in the ratio between commensal and pathogenic strains or growth of new bacterial groups disturb intestinal homeostasis and may perhaps contribute to the pathogenesis or progression of many human diseases including inflammatory bowel diseases (IBD), autoimmune diseases and metabolic disorders [12]. Studies on the influence of dysbiosis on gut health are in progress, however, the multitude and diversity of bacterial strains inhabiting human GI tract make those efforts difficult. So far, neither a single nor a group of bacterial species have been clearly shown to cause the leaky gut syndrome (LGS).

A crucial element of the physical intestinal barrier is formed by epithelium. The small intestine epithelium is composed of a single layer of columnar cells, mainly absorptive enterocytes, but also secretory goblet, Paneth and enteroendocrine cells [13]. To provide the lowest level of permeability for microorganisms, toxins and antigens, and simultaneously enable the influx of ions and solutes, adjacent epithelial cells are merged by the apical junctional complex, which include TJs, adherent junctions (AJs) and desmosomes [14]. TJ proteins consist of claudins (CLDNs), zonula occludins (ZO), and occludins whereas E-cadherin, α-catenin, and β-catenin form AJs [2]. By regulating the transport of ions and molecules across the epithelium, TJs are involved in cell polarity and signaling, therefore, they are an essential component for maintaining intestinal homeostasis. Beyond the epithelium, the lamina propria provides defense based on innate and acquired immunity cells secreting IgA, cytokines, chemokines and mast cell proteases as well as endocrine and secretomotor mechanisms mediated by the enteric nervous system, which result in intestinal propulsive motility [9].

### 1.2. Leaky Gut Syndrome

Variation in the structure of the intestinal barrier due to inflammation, chronic diseases or poor nutrition might lead to impairment of intestinal permeability [15]. According to the LGS hypothesis, intestinal hyperpermeability may enable noxious microorganisms, their toxins and antigens to “leak” into the bloodstream and consequently trigger systemic reactions [16]. Until recently, the LGS was associated with alternative medicine circles however novel evidence indicate its connection between both GI and non-GI diseases. These include celiac disease [17], IBD [18], irritable bowel syndrome (IBS) [19], type 1 [20,21] and 2 diabetes [22], Parkinson disease [23], autism [24] and allergies [25]. 

There are two different pathways of transepithelial transport which if disintegrated may contribute to the pathogenesis of the LGS: paracellular (in between the adjacent cells via dynamic opening and closing of intercellular junctions) and transcellular (through the endothelial cells via endocytosis). Paracellular permeation of molecules is mainly controlled by TJs, which (by forming small pores) regulate the influx of ions and other small molecules (molecular weight <600 Da) through the intestinal wall [26]. Cytokines, intestinal bacteria, or dietary antigens can affect TJs conformation thereby alter intestinal permeability [27]. For instance, interferon γ (INF-γ), released after contact with viruses or certain bacteria, increases intestinal permeability by redistribution of TJ proteins and the rearrangement of the actin cytoskeleton. Tumor necrosis factor α (TNF-α) also influences intestinal permeability via inducing apoptosis of endothelial cells [28]. In addition, interleukin 6 (IL-6) is evidenced to enhanced intestinal permeability by stimulating the expression of CLDN-2 which plays a crucial role in forming TJ pores [29]. Disintegration of transepithelial transport pathways may induce further translocations of noxious factors and as a result of the vicious circle, contribute to progression of many intestinal diseases such as IBD or be one of the pathogenic factors [15]. 

The first human protein displaying regulatory activity on intestinal TJs, called zonulin, was identified in 2000 [30]. Presumably, zonulin activates epidermal growth factor receptor (EGFR) through proteinase activated receptor 2 (PAR_2_) which leads to phosphorylation of TJ proteins and rearrangement of actin filaments and the subsequent displacement of proteins from the junctional complex. As a result, TJ become looser, enhancing intestinal permeability [31]. It is assumed that zonulin contributes to bacteria–host interaction, as increased secretion of this protein was detected in case of exposure to either pathogenic or nonpathogenic strains [32]. Disruptions of intestinal zonulin concentration was observed in patients with celiac disease and IBS indicating its role in the pathogenesis or progression of these diseases [33,34].

There are plenty of environmental factors capable of changing intestinal barrier permeability: physical and psychological stress [35], obesity, alcohol [36], antibiotics and nonsteroidal anti-inflammatory drugs (NSAIDs), food allergens and nutrients itself [37]. It is well known that dietary components may significantly alter GI functions and specifically modulate intestinal barrier integrity. The influence of dietary habits on intestinal physiology is clearly visible in differences in incidence rates of intestinal diseases between industrial and developing countries. Diseases which are linked to intestinal hyperpermeability tend to localize to Westernized countries, where a diet rich in fats and refined carbohydrates predominates [38]. Moreover, several in vivo studies support the connection between a high-fat diet (HFD) and disturbance in the physiology of the GI tract. For instance, Cheng et al. [39] evidenced that HFD exacerbates experimental colitis in a mouse model of IBD. Additionally, a Western-style diet alters intestinal microbiota composition and reduces the concentration of beneficial commensal bacteria including *Bifidobacterium* spp. and *Bacterioides* spp. which contribute to preserving intestinal barrier integrity [40].

This review presents current knowledge about the impact of dietary carbohydrates and lipids on the pathophysiology of LGS. 

## 2. The influence of Dietary Carbohydrates on the Intestinal Barrier

Carbohydrates are classified as one of the three principal classes of macronutrients. They are primarily found in natural food sources, such as cereal products, dry seeds of legumes, fruit, and vegetables. Additional sources of carbohydrates include sugary drinks, sweets, and commercial prebiotics. Depending on the number of sugar units in the chemical structure, they are classified into sugars (mono- and di-saccharides), oligo-, and poly-saccharides. The main dietary monosaccharides are glucose and fructose. A common disaccharide, sucrose, contains equal parts of glucose and fructose. Most other dietary di-, oligo- and poly-saccharides are made up of glucose, fructose, and galactose, to which they may be hydrolyzed during digestion [41].

Saccharides are the main source of energy for both intestinal eukaryotic cells and gut microbiota. The metabolome analysis showed that microbial cells express an enriched spectrum of glycoside hydrolases. As a result, they may also acquire energy from carbohydrates, which are indigestible in humans [42]. These saccharides are fermented into short chain fatty acids (SCFAs), which have a positive impact on the function of intestinal cells [43].

According to many studies, dietary carbohydrates may affect intestinal barrier function. The majority of reports in this field focus on the role of fructose and galacto-oligosaccharides (GOS) in the gut hence here we describe the crucial findings related to those compounds (Table 1).

### 2.1. Fructose as a More Affecting Factor than Glucose 

Numerous animal and human studies showed that the natural monosaccharide found in fruits, fructose, plays a key role in maintaining the continuity of the intestinal wall. Bergheim et al. [44] demonstrated that in fructose-fed mice, both the endotoxin concentration in plasma and the hepatic fat accumulation were significantly increased. These differences were greater as compared to artificial sweetener, glucose, and sucrose. Enhanced endotoxin translocation was confirmed by numerous further studies and may be explained by the fructose-induced dysfunction of TJ proteins. Spruss et al. [45] showed that duodenal samples from fructose-fed mice are characterized by decreased levels of occludin and ZO-1 proteins. Moreover, they observed the overactivation of matrix metalloproteinases, MMP-9 and MMP-13 taking part in post-translational degradation of occludin and decreased level of tissue inhibitor of MMP (TIMP)-1. Interestingly, the negative impact of the fructose on TJs was effectively reduced by metformin. Another study also showed that overuse of fructose decreased the levels of occludin and ZO-1 proteins in mouse colonic samples. Moreover, those observations were similar in mice fed with high-glucose, and high-fat diets [46]. Similar results were seen in studies on fructose-fed rats. The intestinal samples showed decreased levels of TJ proteins: occludin, ZO-1, CLDN-1, and CLDN-4 as well as β-catenin and E-cadherin, desmosome plakoglobin, and α-tubulin [47,48]. It is worth adding that studies on the TJ proteins and defensins mentioned above refer to the expression at the protein level. Other studies showed that, at smaller doses, fructose may also influence the expression at the level of transcription. Nevertheless, the alterations in mRNA levels are mostly not significant [49,50], except for a-defensin 1, occludin and CLDN-2 in the ileum, and CLDN-5 in the colon [51].

The mechanism by which fructose increases intestinal permeability is not entirely understood so far. One hypothesis is linking this simple sugar with inflammasome dysfunction. One of the inflammasome domains, NOD-like receptor family pyrin domain-containing 6 (NLRP6), is involved in maintaining epithelial cells’ integrity. It takes part in the secretion of mucus and the proliferation of epithelial cells [52]. Fructose-fed mice exhibit downregulation of NLRP6 and IL-18, the production of which is associated with NRLP6. These alterations, together with an impaired epithelial barrier, are ameliorated by SCFAs and pioglitazone, which is an antidiabetic drug acting as an agonist of peroxisome proliferator-activated receptor γ [53].

Another possible mechanism of fructose effect on gut barrier integrity involves ethanol-inducible cytochrome P450-2E1 (CYP2E1). This enzyme is primarily expressed in hepatic cells where it is responsible for alcohol metabolism [48,54]. Fructose increases intestinal permeability through interaction with CYP2E1 and this effect is not observed in in the fructose-fed CYP2E1 knockout mice. Moreover, fructose significantly increases the intestinal CYP2E1 expression, which is involved in the alcohol-induced LGS. 

Of note, intestinal samples from fructose-fed rodents are characterized by histopathological changes. Fructose ingested in high doses promotes inflammation, which is shown by high inflammatory cell infiltration [47,50,51]. Another negative change in epithelial morphology is a loss of mucosal thickness [47,51]. However, such a feature is not always observed [50]. Moreover, treatment with fructose increases the villi width in rats [55].

The inhibition of fructose absorption seems to be a promising tool to abolish the negative impact of this compound on the intestinal permeability and other fructose-induced disorders. In the epithelium, fructose is transported across enterocytes mainly by the GLUT-5 transporters in the apical surface, and then by the GLUT-2 expressed on the basolateral membrane [56]. GLUT-5 on Caco-2 cells may be directly inhibited by two phytochemicals: nobiletin and epicatechin gallate. Caco-2 cell line is a human colorectal adenocarcinoma line with no mucus present, used in LGS studies as a model of the intestinal epithelial barrier. As a result, the fructose uptake is reduced to the levels below 60% of the control [57]. An in vivo study on rats fed with fructose and dietary polyphenol—chrysin— showed alterations in GLUT-5 expression leading to its lower activity [55]. Unfortunately, even if the absorption of fructose is suppressed, the fructose-induced LGS may continue to develop due to delayed dysbiosis [55]. Of note, dysbiosis triggered by high consumption of fructose is reported by numerous studies although those reports do not provide consistent results about exact changes in gut microbiota profile [48,50,51,53]. This could be explained by the fact that the development of dysbiosis is a multifactorial process. Alterations in the intestinal microbiota are also dependent on the use of antibiotics, stress and dietary composition [58] and microelements, such as copper level in the diet [59]. 

The evidence of fructose-induced impairment of epithelial permeability in animal experiments consequently led to human studies. Kuzma et al. [60] did not confirm the negative properties of fructose on the intestinal barrier. They observed that plasma levels of zonulin and LPS-binding protein (LBP), a marker of endotoxemia did not differ in individuals undergoing eight-day periods of high-fructose, high-fructose corn syrup, or high-glucose diet, which were separated by washout periods. Another study confirmed that fructose did not alter the LBP plasma concentrations [61]. On the other hand, it was showed that, fructose, but not glucose, significantly increased the plasma concentration of endotoxins after short, 3 days exposure to high doses of those carbohydrates.

Described animal and human studies suggest that the current state of knowledge regarding the role of glucose in LGS is insufficient, and the data comparing the fructose with glucose are not consistent in all aspects. However, there is a lack of studies suggesting that glucose influences the intestinal permeability to a higher degree than fructose does. To expand the knowledge about the exact role of the glucose in the LGS, studies on diabetes mellitus seem to be a promising field of research. It is well established that chronic hyperglycemia is associated with a range of GI complications, such as impaired motility or morphological changes in small intestine. Moreover, increased intestinal permeability with zonulin dysregulation is observed in diabetic patients [62].

### 2.2. Complex Carbohydrates in Prebiotics

Complex carbohydrates are made up of more than two sugar units. Some of them are found in natural or commercial prebiotics, which protect against dysbiosis. One of the natural prebiotics is dietary fiber (DF) found in plants. DF contains soluble and insoluble forms, which are composed of polysaccharides, such as soluble inulin and insoluble cellulose, and non-polysaccharide compounds. It was showed that, in humans, DF consumption has beneficial effects on the intestinal permeability. The increase in DF (both soluble and insoluble) intake from 19 to 29 g per day in a half-year trial resulted in significantly decreased serum zonulin levels [63]. DF is indigestible by endogenous enzymes, however, gut microbiota can convert it into small metabolites, such as SCFAs which are shown to be involved in the maintenance of the intestinal barrier function. Among the different SCFAs from DF fermentation, the butyrate seems to be a key factor protecting from leaky gut [64,65], and is mainly produced from soluble DF [66]. 

Other saccharides that maintain proper intestinal function are lactulose, arabinose, inulin-type fructans, fructo-oligosaccharides (FOS) and GOS [67,68]. All of them support the growth of beneficial bacteria; however, some studies suggest that GOS naturally found in human breast milk directly interact with intestinal cells. The in vitro treatment with GOS protects Caco-2 cells from a decrease in transepithelial electrical resistance (TEER) as well as restores proper activities of CLDN-3 and chemokine CXC motif ligand 8 (CXCL8) after exposure to deoxynivalenol (DON), a natural toxin affecting epithelium. TEER is a widely accepted quantitative technique used to measure the integrity of TJ dynamics in cell culture models of endothelial and epithelial monolayers. TEER values are strong indicators of the integrity of the cellular barriers; it is considered that elevated TEER indicates an increase, whereas lower TEER indicates a decrease in barrier integrity. Additionally, this treatment accelerates the recovery of TJs after calcium deprivation in the same cell line [69]. Another study on Caco-2 cells exposed to DON compared the effects of GOS in different forms, FOS and inulin [70]. The research examined the preventive effect of those saccharides on disruption of monolayer integrity and subsequent CXCL8 release. Non-purified GOS was the most effective in this test. In the in vivo part of the former study, authors demonstrated beneficial effects of GOS in mice exposed to DON. Investigation of intestinal samples obtained from those animals showed that GOS suppresses the overexpression of CLDN-3, CXCL1 and CXCL2. Moreover, mice pretreated with GOS were prevented from DON-induced morphologic alterations in the small intestine [68]. A study on GOS-supplemented piglets showed up-regulation of TJ proteins in different parts of the GI tract. The alterations of mRNA expression and protein levels depended on the duration of exposure to GOS and exact place of sampling. Nevertheless, among examined proteins, CLDN-1, ZO-1 and ZO-2 were the up-regulated the most by GOS [71].

Krumbeck et al. [72] investigated the role of *Bifidobacterium* and GOS on intestinal barrier function in humans. One of the research groups including 20 obese individuals treated with high-GOS diet for 3 weeks showed significantly decreased intestinal permeability. Patients underwent examination of post-aspirin sucralose and lactulose excretions to urine to determine the permeability of the gut wall.

## 3. The Influence of Dietary Lipids on the Intestinal Barrier

Lipids are critical components of every living cell. They warrant the integrity of the bilayer structure of the cell membrane. Many reports proved the influence of lipids on gut permeability through alteration of TJs gene expression and histone acetylation [73,74]. An in vivo study on C57BL/6J mice demonstrated that the proximal colon of mice fed with high saturated fat diet (HSFD) exhibited decreased TEER and mRNA expression of ZO-1 as compared to mice fed with the control diet suggesting increased gut permeability. Moreover, HSFD mice had altered gut microbiota profile, including a significantly higher number of *Firmicutes* and lower of *Bacteroides*. TEER of the proximal colon was positively correlated with the abundance of *Lactobacillus*, but negatively associated with *Oscillibacter*. Interestingly, increased *Oscillibacter* abundance was also associated with a reduction in the mRNA expression of ZO-1 [75]. 

### 3.1. Effect of Short Chain Fatty Acids on Gut Permeability

Short chain fatty acids (SCFAs) including acetate, butyrate and propionate are the end products of anaerobic bacterial fermentation of DF in the colon [76]. The intake and composition of DF modulate production of SCFAs, especially butyrate in the large intestine. SCFAs are principal energy source for colonocytes and they are also involved in many biological processes in the gut, such as cellular differentiation, growth arrest, and apoptosis of colonic epithelial cells [77]. 

Many studies indicated that butyrate and propionate induce a “tightening” effect on TJ permeability in Caco-2 cell line. Butyrate and propionate decreased the fluorescein sulfonic acid (FS) permeability ratio and increased TEER ratio. The effect of butyrate on FS and TEER ratios was stronger than those of propionate. On the other hand, acetate did not significantly influence those parameters [74,78]. A potential mechanism of SCFAs effect on gut permeability was investigated by Ohata et al. [74]. They focused on the effect of lipoxygenase (LOX) and cyclooxygenase (COX) inhibitors on intestinal barrier and on changes in the expression of those enzymes induced by SCFAs in Caco-2 cells. Results showed that only LOX inhibitor significantly reversed the effect of butyrate on gut permeability, whereas both LOX and COX inhibitors partly reduced the effect of propionate. On the other hand, butyrate increased LOX expression as well as the level of LOX product, hydroxyeicosatetraenoic acid and trichostatin A, which is a histone deacetylase (HDAC) inhibitor. In summary, SCFAs are engaged in gut permeability alterations via LOX activation and histone acetylation. Thus, butyrate is responsible for inhibiting histone deacetylation that subsequently leads to hyperacetylation [74]. Another possible mechanism involves the activation of the AMP-activated protein kinase (AMPK). Peng et al. [79] found that butyrate increased AMPK activity and is involved in TJ protein reorganization as well as significantly increased TEER in the Caco-2 monolayers. In detail, butyrate increased the amount of phosphorylated AMPK in a time-dependent manner. Furthermore, compound C, a specific AMPK inhibitor detained the butyrate-induced upregulation of AMPK. Elamin et al. [80] confirmed that SCFAs, including butyrate, propionate and acetate activate AMPK and as a consequence, ameliorate ethanol-induced intestinal barrier dysfunction in Caco-2 cell monolayers. Recent studies in another cell line, E12 human colon cells, demonstrated that sodium-butyrate at the concentration of 1–10 mM significantly improved the epithelial barrier function, whereas higher concentrations (50–100 mM) showed no beneficial effect [81]. Interestingly, previous study by Peng et al. [82] using Caco-2 cells showed that butyrate reduced barrier function at high concentrations. In both studies, butyrate did not influence the expression of intercellular junction proteins, such as ZO-1 in E12 cells or occludin, CLDN-1 and 4 and ZO-1 in Caco-2 cells. Furthermore, in an isolated vascularly perfused rat colon model butyrate at the concentration 5 mM induced increased colonic mucin secretion and thereby, indirectly *MUC2* gene expression [83]. In vivo study with pigs fed with a fat-rich diet which induces alterations in large intestinal SCFA production showed minor influence of this treatment on parameters related to intestinal barrier function, including elevated level of *MUC2* gene expression. However, the relationship between *MUC2* expression and luminal concentration of butyrate was ambiguous. TJ proteins were also not altered [84]. Additionally, in vivo rodent studies showed similar obscure relation between increased expression of *MUC2* and luminal butyrate [85]. 

In summary, there is a wealth of evidence supporting the beneficial effect of butyrate and other SCFAs on TJ permeability and enhancement of intestinal barrier (Table 2). Mechanism of this effect is related to elevated level of LOX and/or AMPK; however, the role of TJ proteins in those effects is still investigated.

### 3.2. Effect of Long Chain Fatty Acids on Gut Permeability

Long chain fatty acids (LCFAs), which are consumed in the diet may induce biological responses and affect intestinal permeability. The *n*-3 and *n*-6 polyunsaturated fatty acids (PUFAs) are involved in many physiological processes, such as immunological reactions as well as the pathophysiology of several disorders, e.g., LGS and IBD [86,87]. The influence of PUFAs on TJ permeability of the gut has also been reported. However, there are some contradictory points. Howie et al. [88] discovered that decosahexaenoic acid (DHA, C22:6 *n*-3) protects the small intestine of mice from the increased permeability induced by methotrexate. On the contrary, there is evidence that a water-in-oil-in-water emulsions incorporating C18 unsaturated fatty acids or DHA were valuable carriers for improving the colonic absorption of poorly absorbable drugs, suggesting increased intestinal absorption. Nevertheless, TEER was not changed by the incorporation of various fatty acids in emulsions indicating that this effect was not related to an increase in gut permeability [89].

The effect of C18, including α-linolenic acid (ALA, C18:3 *n*-3), linoleic acid (LA, C18:2 *n*-6), or oleic acid (OA, C18:1 *n*-9) and C20, including eicosapentaenoic acid (EPA, C20:5 *n*-3) and arachidonic acid (AA, C20:4 *n*-6) on TJ permeability was investigated in intestinal monolayer Caco-2 cells [90]. Every acid was added to culture medium at the concentration of 200 µM for 24 h. After incubation with LCFA, FS permeability, TEER, lactate dehydrogenase release and ultrastructure of cells were investigated. Results showed that only EPA and ALA enhanced paracellular permeability (measured by TEER) and permeability of lipophobic small molecules. In detail, EPA significantly improved FS permeability up to 3.0 ± 1.6-fold and lowered TEER down to 0.59 ± 1.2-fold vs. control without cell injury. The same group of researchers later showed that γ-linolenic acid (GLA; C18:3 *n*-6) or DHA increased FS permeability 8.7- and 1.4-fold, respectively, and lowered TEER 0.52- and 0.73-fold, respectively, versus control without cell injury. COX and LOX inhibitors enhanced the effect of GLA but not DHA [91]. Furthermore, Willemsen et al. [92] showed that AA, EPA, DHA and (to a lower extent) GLA enhanced basal TEER and strongly reduced IL-4-mediated permeability suggesting that those PUFAs support barrier integrity. Moreover, recent studies showed that EPA and DHA improve intestinal barrier function as indicated by higher TEER and lower FITC-dextran flux as well as increased proportions of TJ proteins located in the plasma membrane [93] (Table 2). In fact, the addition of lipase, which was used to simulate in vivo digestion and release fatty acids from the glycerol backbone, increased basal TEER in T84 cells in a dose-dependent manner.

The mechanism of changing the TJ permeability by LCFAs was investigated using COX inhibitor, indomethacin, LOX inhibitors, NDGA or AA861, and an antioxidant, BHT [90]. Authors discovered that the effect of EPA on TJ was reversed by AA861. Indomethacin partially inhibited the effect of EPA on FS permeability. These results suggest that the effect of EPA on TJ is depend on COX and LOX activity. In addition, the effect of BHT, in combination with EPA was investigated to evaluate the influence peroxidation products generated from EPA. BHT did not change TEER or FS permeability itself nor did it influence the effect of EPA suggesting that the oxide did not alter TJ permeability. Another mechanism of TJ alteration involved protein kinase C (PKC). It was showed that PKC antagonists facilitate the changes mediated by GLA and DHA suggesting that PKC also may be engaged in the mechanism of leaky gut [91]. 

In humans, in a cross-sectional study, Mokkala et al. [97] investigated whether gut microbiota and diet affect serum zonulin concentration in 100 overweight Finnish women in early pregnancy. They found that the richness and composition of the gut microbiota as well as the intake of *n*–3 PUFAs, fiber, and a range of vitamins and minerals were associated with low serum zonulin concentration. Furthermore, Genser et al. [94] demonstrated, using immunofluorescence, TJ damage in the jejunal epithelium of obese patients indicated by a reduction of occludin and tricellulin. Moreover, they investigated whether the barrier impairment could be exacerbated by dietary lipids. The jejunal permeability after the lipid load was two-fold higher in obese patients as compared to non-obese controls; this suggests that intestinal barrier defects occur in obesity and may be indirectly associated with lipid consumption. 

### 3.3. Other Lipids

Medium chain fatty acids (MCFAs), such as capric acid (C10) and lauric acid (C12) induce a rapid increase in epithelial permeability to the hydrophilic marker, FS in Caco-2 cells. However, capric acid, not lauric acid causes redistribution of the TJ proteins ZO-1 and occluding suggesting that these two MCFAs have partially different mechanism of action [98]. Importantly, food additives also may act as permeation enhancers. Recently, Glynn et al. [99] demonstrated the additive effect of the dietary surfactants solanine and chaconine, perfluorooctate sulfonic acid and sucrose monolaurate on TJ integrity leading to disruption of the intestinal barrier as measured by increased TEER in Caco-2 cell monolayers. 

Notably, phosphatidylcholine (PC), a major surface-active phospholipid (PL) is a component of the intestinal mucus barrier. Olson et al. [100] evidenced that exogenous PC supplementation improves intestinal barrier defense against *Clostridium difficile* toxins, A and B in vitro. Using a common method with FITC-dextran, researchers observed that PC administration reduced intestinal epithelial cells permeability to baseline level in the non-mucus-producing and mucus-producing HT-29 cells. Moreover, actin staining showed that pretreatment with PC protects F actin cytoskeleton against toxin A. PC also reduced the level of TNFα and IL-6 as compared to control group. Additionally, in vivo study showed that PC protects against increases in gastric and ileal tissue permeability in the model of LPS-induced GI injury [101]. 

PLs and PC are also used to improve drug absorption [101,102]. The effect of DHA-enriched PC and EPA-enriched PC on intestinal permeability had been investigated. Hossain et al. [103] demonstrated that DHA- and EPA-enriched PC enhance the permeability and transport across monolayer of Caco-2. In this study TEER decreased down to 24% and 27% with 50 and 100 μM DHA-enriched PC, and to 25% and 30% with 50 and 100 μM EPA-enriched PC, respectively. Furthermore, Konishi et al. [102] investigated the transport and uptake of liposomes composed of PC, phosphatidylserine (PS), and sulfoquinovosyl diacylglycerol (SQDG) in small intestinal epithelial cell models. Liposomes containing PS exhibited higher uptake by both Caco-2 cell and M cell monolayers as compared to PC. SQDG-containing liposomes exhibited higher transport through M cell but not Caco-2 monolayer, while its uptake was higher in both types of monolayers. Importantly, researchers evidenced that PC/SQDG could untighten the TJs of small intestinal epithelial cell monolayers [102].

In summary, exogenous administration of PC may decrease gut permeability caused by *Clostridium difficile* toxin, however, it is also used to increase drug absorption given its ability to reduce TEER, suggesting elevated intestinal permeability.

## 4. Conclusions and Future Perspective

**Figure 1:**
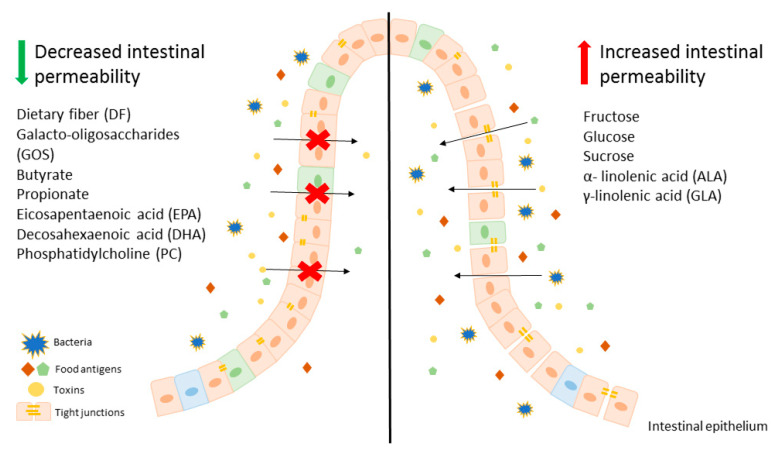
An overview on the effect of various components of diet on intestinal epithelium permeability.

Modification of diet, especially in terms of carbohydrates and lipids composition, may beneficially affect intestinal permeability, leading to an improvement of LGS. It is worth mentioning that there are several studies showing that fructose is one of the key carbohydrates involved in regulation of the intestinal permeability and causes mainly harmful effects. On the other hand, free fatty acids seem to have a beneficial effect on gut permeability, for instance SCFAs, mainly butyrate at appropriate concentrations may lead to the reduction of intestinal permeability, which is beneficial in LGS. In contrast, LCFAs, including *n*-3 and *n*-6 PUFAs have unclear properties. Some of those behave as components untightening and tightening intestinal membrane. These evidences suggest that modification of a diet can be an adjunct to convectional, pharmacological therapy for patients with LGS and other disorders characterized by leaky gut. A potential beneficial diet would be based on avoidance of food products, such as fruits abundant in fructose as well as oils containing ALA and GLA. On the other hand, patients with LGS should consume a larger amount of dietary fiber (Figure 1).

To conclude, the development of novel therapeutic approaches for patients with LGS and other disorders with accompanying impairment of the gut–blood barrier aims to design personalized nutrition therapy to decrease carbohydrate- or lipid-induced intestinal leakage. However, there are still many questions about LGS and dietary components, therefore further research is needed.

## Figures and Tables

**Table 1:** The effects of carbohydrates (fructose and GOS) on intestinal permeability and their influence on proteins involved in intestinal barrier integrity. Results obtained from cell, animaland human studies.

Type of Study	Intestinal Permeability; Measurement Method	Levels of TJ Proteins	Levels of Other Proteins Directly Related to Cells Integrity	References
**Fructose**				
in vitro: T84 cells	↑; TEER *, FITC-dextran concentration	↓ ZO-1	-	Cho et al. [48]
in vitro: HT-29 cells	↑; FITC-dextran concentration	↓ Occludin (mRNA)	-	Kawabata et al. [50]
in vivo: mouse	↑; endotoxemia	-	-	Bergheim et al. [44]
in vivo: mouse	↑; endotoxemia	↓ Occludin ↓ ZO-1	↑ MMP-9 (mRNA) ↑ MMP-13 (mRNA) ↓ (TIMP)-1	Spruss et al. [45]
in vivo: mouse	↑;endotoxemia, FITC-dextran concentration	↓ Occludin ↓ ZO-1	-	Do et al. [46]
in vivo: rat	↑;LBP, FITC-dextran concentration	↓ ZO-1	-	Seki et al. [47]
in vivo: mouse and rat	↑; endotoxemia, LPS (mouse and rat), FITC-dextran concentration (only mouse)	↓ ZO-1 ↓ Occludin ↓ CLDN-1 ↓ CLDN-4	↓ β-catenin ↓ E-cadherin ↓ Desmosome plakoglobin ↓ α-tubulin	Cho et al. [48]
in vivo: mouse	↑; endotoxemia, NS; FITC-dextran and PEG400 concentrations, lactulose:mannitol ratio	↓ Occludin (mRNA) ↓ CLDN-2 (mRNA) ↓ CLDN-5 (mRNA)	↓ α-defensin 1 (mRNA)	Volynets et al. [51]
in vivo: mouse	↑; endotoxemia, FITC-dextran concentration	↓Occludin ↓ ZO-1	-	Li et al. [53]
in vivo: human	↑; endotoxemia, NS; LBP	-	-	Nier et al. [61]
**GOS**				
in vitro: Caco-2 cells	Prevents from: ↑; TEER *	-	-	Akbari et al. [70]
in vitro: Caco-2 cells	Prevents from: ↑; TEER *, FITC-dextran and lucifer yellow concentrations	Prevents from: ↓ CLDN-3 (protein) ↑ CLDN-3 (mRNA)	-	Akbari et al. [69]
in vivo: mouse	NS; FITC-dextran concentration	Prevents from: ↑ CLDN-2 (mRNA) ↑ CLDN-3 (mRNA)	-	
in vivo: human	↓; 24 h collection of sucralose and lactulose excretions to urine NS; LPS, LBP	-	-	Krumbeck et al. [72]

Abbreviations: TJ—tight junction, ZO—zonula occludin, MMP—matrix metalloproteinase, TIMP—tissue inhibitor of matrix metalloproteinases, LPS—lipopolysaccharide, LBP—LPS-binding protein FITC—fluorescein isothiocyanate, TEER—transepithelial electrical resistance, T84; HT29—human colon cancers cell lines, CLDN—claudin, PEG400—polyethylene glycol 4000, GOS—galacto-oligosaccharide, NS—not significant/no change, ↑—increased, ↓—decreased. * The increase in permeability is indicated by decreased values of TEER.

**Table 2:** The effect of fatty acids on intestinal permeability markers in in vitro and in vivo studies.

Component	Fluorescein Sulfonic Acid Permeability In Vitro	Transepithelial Electrical Resistance In Vitro	Measurements In In Vivo Studies	Effect	References
**Short Chain Fatty Acids**					
acetate	NS	↑	↓ blood-to lumen clearance of ^51^Cr-EDTA	tightening	Elamin et al. [80] Wan Saudi et al. [94]
butyrate	↓	↑	increased colonic mucin secretion	tightening	Peng et al. [79] Elamin et al. [80] Nielsen et al. [81] Peng et al. [82] Barcelo et al. [83]
propionate	↓	↑	↓ blood-to lumen clearance of ^51^Cr-EDTA	tightening	Elamin et al. [78] Wan Saudi et al. [95]
**Long Chain Fatty Acids**					
oleic acid (OA)	NS	NS	-	-	Usami et al. [90]
linolenic acid (LA)	NS	↓	-	slightly untightening	Usami et al. [90]
α-linolenic acid (ALA)	↑	↓	-	untightening	Usami et al. [90]
arachidonic acid (AA)	NS	↓/↑	-	slightly untightening/tightening	Usami et al. [90] Willemsen et al. [92]
eicosapentaenoic acid (EPA)	↑	↓/↑	-	untightening/tightening	Usami et al. [90] Xiao et al. [93] Willemsen et al. [92]
γ-linolenic acid (GLA)	↑	↓	-	untightening	Usami et al. [91]
decosahexaenoic acid (DHA)	↑	↓/↑	strong insulin permeability enhancement effect	untightening/tightening	Usami et al. [91] Willemsen et al. [92] Xiao et al. [93] Onuki et al. [96]

Abbreviations: AA—arachidonic acid, ALA—α-linolenic acid, DHA—decosahexaenoic acid, EPA—eicosapentaenoic acid, ^51^Cr-EDTA—^51^chromium-labeled ethylenediamine tetraacetic acid, GLA—γ-linolenic acid, LA—linolenic acid, NS—not significant/no change, OA—oleic acid, ↑—increased, ↓—decreased.

## Abbreviations

^51^Cr EDTA	^51^chromium-labeled ethylenediamine tetraacetic acid
AA	arachidonic acid
AJs	adherent junctions
ALA	α-linolenic acid
CLDNs	claudins
COX	cyclooxygenase
CYP2E1	cytochrome P450-2E1
CXCLs	C-X-C motif chemokine ligands
DF	dietary fiber
DHA	docosahexaenoic acid
DON	deoxynivalenol
EGFR	epidermal growth factor receptor
EPA	eicosapentaenoic acid
FITC	fluorescein isothiocyanate
FS	fluorescein sulfonic acid
FOS	fructo-oligosaccharide
GI	gastrointestinal
GLUTs	glucose transporters
GOS	galacto-oligosaccharide
HDAC	histone deacetylase
HFD	high fat diet
HSFD	high saturated fat diet
IBD	inflammatory bowel disease
IBS	irritable bowel syndrome
IL	interleukin
INF-γ	interferon γ
LA	linoleic acid
LBP	LPS-binding protein
LCFAs	long chain fatty acids
LGS	leaky gut syndrome
LPS	lipopolysaccharide
LOX	lipoxygenase
MCFAs	medium chain fatty acids
MMP	matrix metalloproteinase
NSAIDs	nonsteroidal anti-inflammatory drugs
OA	oleic acid
PAR	proteinase activated receptor
PC	phosphatidylcholine
PEG400	polyethylene glycol 4000
PL	phospholipids
PS	phosphatidylserine
PUFAs	polyunsaturated fatty acids
SCFAs	short chain fatty acids
SQDG	sulfoquinovosyl diacylglycerol
TEER	transepithelial electrical resistance
TIMP	tissue inhibitor of matrix metalloproteinases
TJ	tight junction
TNFα	tumor necrosis factor α
ZO	zonula occludin
